# Radiation Dose Management in Pediatric Brain CT According to Age and Weight as Continuous Variables

**DOI:** 10.3390/tomography8020079

**Published:** 2022-04-01

**Authors:** Yusuke Inoue, Hiroyasu Itoh, Anri Waga, Ryosuke Sasa, Kohei Mitsui

**Affiliations:** 1 Department of Diagnostic Radiology, Kitasato University School of Medicine, Sagamihara 252-0374, Japan; irnag.waa@icloud.com (A.W.); km19901004@gmail.com (K.M.); 2 Department of Radiology, Kitasato University Hospital, Sagamihara 252-0375, Japan; hiroyasu@kitasato-u.ac.jp (H.I.); r-sasa@kitasato-u.ac.jp (R.S.)

**Keywords:** computed tomography, radiation dose, pediatrics, brain, diagnostic reference level

## Abstract

The diagnostic reference levels (DRLs) for pediatric brain computed tomography (CT) are provided for groups divided according to age. We investigated the relationships of radiation dose indices (volume CT dose index and dose length product) with age and weight, as continuous variables, in pediatric brain CT. In a retrospective analysis, 980 pediatric brain CT examinations were analyzed. Curve fitting was performed for plots of the CT dose indices versus age and weight, and equations to estimate age- and weight-dependent standard dose indices were derived. Standard dose indices were estimated using the equations, and the errors were calculated. The results showed a biphasic increase in dose indices with increasing age and weight, characterized by a rapid initial and subsequent slow increase. Logarithmic, power, and bilinear functions were well fitted to the plots, allowing estimation of standard dose indices at an arbitrary age or weight. Error analysis suggested that weight was mildly better than age and that the best results were obtained with the bilinear function. Curve fitting of the relationship between CT dose indices and age or weight facilitates the determination of standard dose indices in pediatric brain CT at each facility and is expected to aid the establishment and application of the DRLs.

## 1. Introduction

Radiation exposure in medical imaging increases the risk of cancer induction and is a major concern in clinical medicine [1,2,3,4,5]. Consideration of radiation dose is especially important in pediatric patients because children are more sensitive to ionizing radiation and have a longer life expectancy than adults [6]. In children, computed tomography (CT) is mainly utilized in the evaluation of abnormalities of the head. Due to the high radiation dose required for CT, concerns have been raised regarding the incidence of brain tumors in children who previously underwent brain CT [1,2,3]. Consequently, optimization of the radiation dose is a priority in pediatric brain CT [7,8,9,10].

Management of the radiation dose is required for the optimization of imaging protocols, as well as for the detection and prevention of examinations with excessive radiation exposure. In CT, two radiation dose indices—the volume CT dose index (CTDIvol) and dose length product (DLP)—are used in radiation dose management. CTDIvol is an index of absorbed dose in the scan region, while DLP is an integral of CTDIvol over the longitudinal scan range and reflects total radiation exposure in an imaging series. The diagnostic reference level (DRL) is recommended as an optimization tool in medical imaging [11,12]. DRL is an advisory value to recognize the need for further optimization at an imaging facility and is set for CTDIvol and DLP in CT. To establish the DRL value, the standard radiation dose used at a facility is determined, usually as the median value of the dose indices obtained in clinical practice. A dose survey is performed in a country or region, and the DRL is commonly defined as the 75th percentile value of the dose distribution. After the establishment of the DRL, each facility compares its standard dose with DRL. A standard dose higher than the DRL implies that the standard dose is relatively high in the country or region and that dose reduction, while preserving clinical utility, should be considered with high priority.

The appropriate imaging parameters and radiation dose in CT depend on the size of the imaging object and thus differ between children and adults. Furthermore, because children steadily grow, they should not be categorized into a single group for dose assessment. Consequently, pediatric DRLs are established in children grouped according to either age (for example, 1–5-year-old group) or body weight (for example, 5–15 kg group) [12,13], and the grouping method can be a matter. Weight better reflects body size than age, and therefore, weight-based grouping is recommended to establish the DRL in body CT. However, age-based grouping is recommended for brain CT because weight does not well reflect head size. Furthermore, dividing children into groups based on weight or age can complicate the evaluation of radiation dose. Due to its high radiation dose, the number of CT examinations performed on children is small in most facilities, and division into groups further decreases the number in each group, possibly disturbing the determination of a median value for each group with acceptable statistical validity. Instead of grouping, curve fitting of the relationship between the dose index and weight as a continuous variable is proposed for body CT [14,15] and recommended to overcome the sample size problem [12,13].

In this study, we retrospectively analyzed a large amount of brain CT data obtained at a single facility and evaluated the relationships of CTDIvol and DLP with age and weight as continuous variables. Curve fitting was performed using logarithmic, power, and bilinear functions, and equations to estimate standard dose indices depending on age or weight were determined. The validity of age- and weight-based estimations were then assessed. Our aim was to establish the method to determine standard CT dose indices at each facility appropriate for radiation dose management in pediatric brain CT.

## 2. Materials and Methods

### 2.1. Subjects

**Figure 1:**
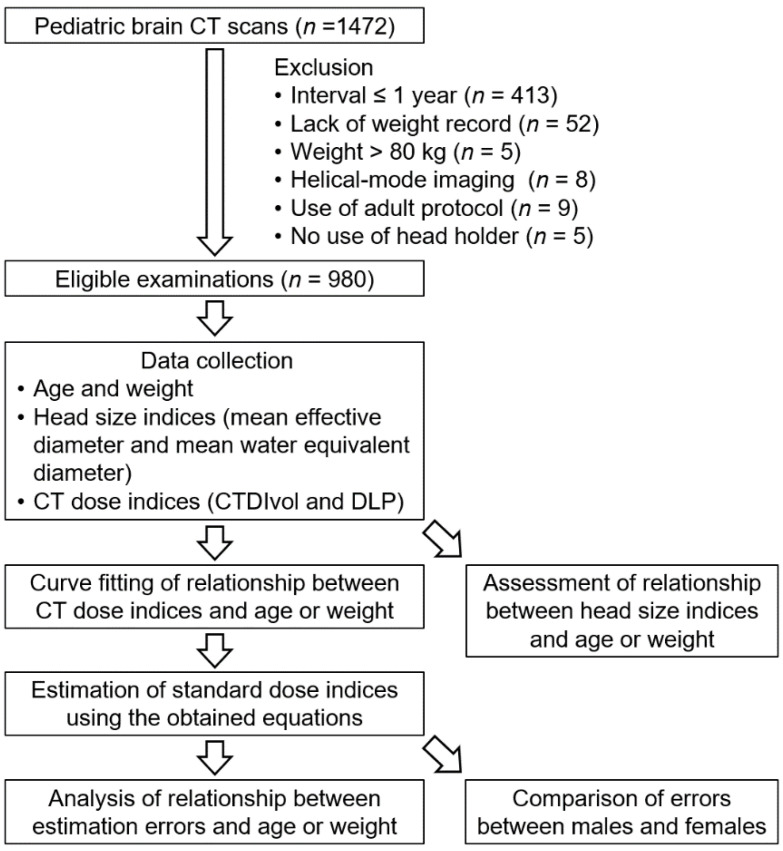
Data selection and outline of the study design.

This retrospective analysis was based on 980 brain CT examinations (544 males and 436 females) performed in children aged < 15 years at a single institution. For patients who underwent more than one CT examination, data from the scans performed at an interval > 1 year were included. The exclusion criteria were as follows: lack of records of body weight, weight > 80 kg, imaging performed in helical mode, erroneous use of an adult CT protocol, and placement of the head on the imaging table or trauma board instead of on the head holder (Figure 1). Kitasato University Medical Ethics Organization approved this study (B20–114), and the need for informed consent was waived due to its retrospective design.

Patient age was calculated as the difference in years, months, and days between the date of birth and the date of the examination, and was expressed in years using a real number. Usually, 1 year of age means that the interval between birth and the examination was ≥1 year but <2 years; however, this means that in this study, the examination was performed on the day just 1 year after birth. For logarithmic transformation, age was regarded as 0.003 years, corresponding to 1 day, when CT was performed on the day of birth.

### 2.2. Imaging Procedures

All CT examinations were performed on one of two 64-detector row CT scanners with the same specifications (Optima CT 660 Discovery Edition; GE Healthcare, Milwaukee, WI, USA). The patient’s head was placed on the head holder, and posteroanterior and lateral scout images were obtained to plan the scan range. Axial CT images parallel to the orbitomeatal line were acquired and covered the lower margin of the posterior fossa and the top of the brain.

To adjust radiation exposure for each patient and for each location, tube current was modulated using automatic exposure control software 3D mA modulation, consisting of Auto mA and Smart mA, preinstalled on the scanners. This software automatically determines the tube current of the X-ray generator depending on X-ray attenuation by the imaging object, as assessed on the scout image. In larger patients, tube current and consequently radiation exposure increase to compensate for stronger attenuation and keep the image quality constant. Since a single scout image obtained just before CT planning is used to assess attenuation, the lateral scout image was used in this study. In 3D mA modulation, the noise index, minimum current, and maximum current are set by the user. The noise index represents the noise level of CT images reconstructed using filtered backprojection; at our facility, it was set at 4 irrespective of patient age. The maximum and minimum current values define the upper and lower limits of tube current, respectively. In this study, the maximum current was high and the minimum current was low, so there was no effect of these parameters on tube current modulation. Additionally, organ dose modulation, which reduces radiation exposure from the anterior direction, was applied over the orbit to decrease the radiation dose to the eye lens. Other imaging parameters were as follows: axial mode; tube voltage, 120 kV; rotation time, 1 s; beam width, 10 mm; slice thickness, 5 mm; and slice interval, 5 mm.

### 2.3. Data Analysis

The mean effective diameter and mean water equivalent diameter for each CT image set were determined using a radiation dose management system Radimetrics (Bayer Medical Care Inc., Indianola, PA, USA). The effective diameter is a simple geometric mean of the anteroposterior and lateral diameters [16]. The water equivalent diameter is a more advanced index reflecting X-ray attenuation and is determined considering differences in attenuation strength among different tissues [17]. Even if the effective diameter is identical, the water equivalent diameter will be larger for sections containing a larger amount of bone. The effective diameter was determined from the scout images and the water equivalent diameter from the CT slice. Mean values for each image set were calculated by averaging through the longitudinal scan range and were compared with age and weight.

CTDIvol and DLP provided by the CT scanner automatically were recorded. Most examinations were performed in a single phase, and the dose indices represent radiation exposure in a single imaging series. When plain and postcontrast images were acquired, data from plain images were used for analysis.

The relationships between the CT dose indices and age or weight were evaluated. CTDIvol and DLP were plotted against age and weight, and logarithmic, power, and bilinear functions were then fitted to the plots. When fitting a bilinear function to the dose–age plots, linear fitting was performed separately for two age groups (<1.5 and ≥1.5 years). In weight-based analysis, two weight groups (<15 and ≥15 kg) were considered.

Standard dose indices were estimated depending on age or weight by substituting the values thereof into the obtained equations. For the bilinear function, dose indices were calculated using two linear equations, irrespective of age or weight, with the lower value selected as the final value. The error (%) was defined as (estimated value − actual value)/(actual value) × 100. The relationships of the error with age and weight were evaluated. In the age-based analysis, the mean and standard deviation (SD) of the error were calculated for the following six age groups: 0–<0.5, 0.5–<1, 1–<2, 2–<5, 5–<10, and 10–<15 years. In the weight-based analysis, six weight groups were defined: 0–<5, 5–<10, 10–<15, 15–<20, 20–<40, and 40–<80 kg. Moreover, the errors of CTDIvol and DLP estimated using bilinear functions were compared between male and female patients.

### 2.4. Statistical Analysis

Values are presented as mean ± SD. Curve fitting was performed using Microsoft Excel (Microsoft Corp., Redmond, WA, USA). The Wilcoxon rank-sum test was performed using R software to compare the error between male and female patients. A *p* value < 0.05 was deemed statistically significant.

## 3. Results

The mean values for the parameters of interest were as follows: age, 5.5 ± 4.7 years; body height, 101.6 ± 33.8 cm; body weight, 19.7 ± 14.9 kg; mean effective diameter, 13.0 ± 1.7 cm; mean water equivalent diameter, 13.8 ± 2.0 cm; CTDIvol, 21.6 ± 4.6 mGy; and DLP, 300.8 ± 87.1 mGy∙cm.

**Figure 2:**
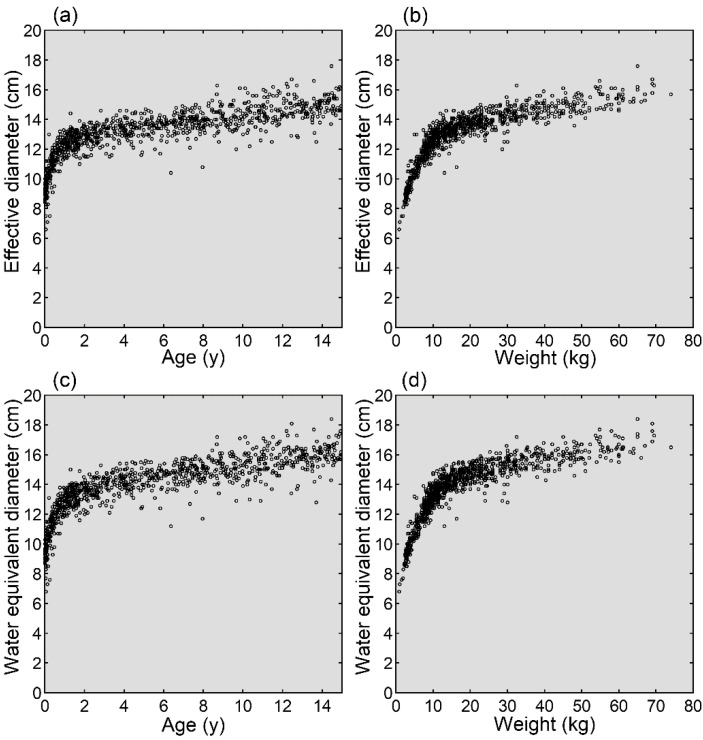
Growth of the head. Mean effective diameters are plotted against age (**a**) and weight (**b**). Mean water equivalent diameters are plotted against age (**c**) and weight (**d**).

The mean effective diameter and mean water equivalent diameter increased with increasing age and weight (Figure 2). A rapid initial increase was observed approximately up to 1.5 years of age or 15 kg of weight, followed by a slow continuous increase.

**Figure 3:**
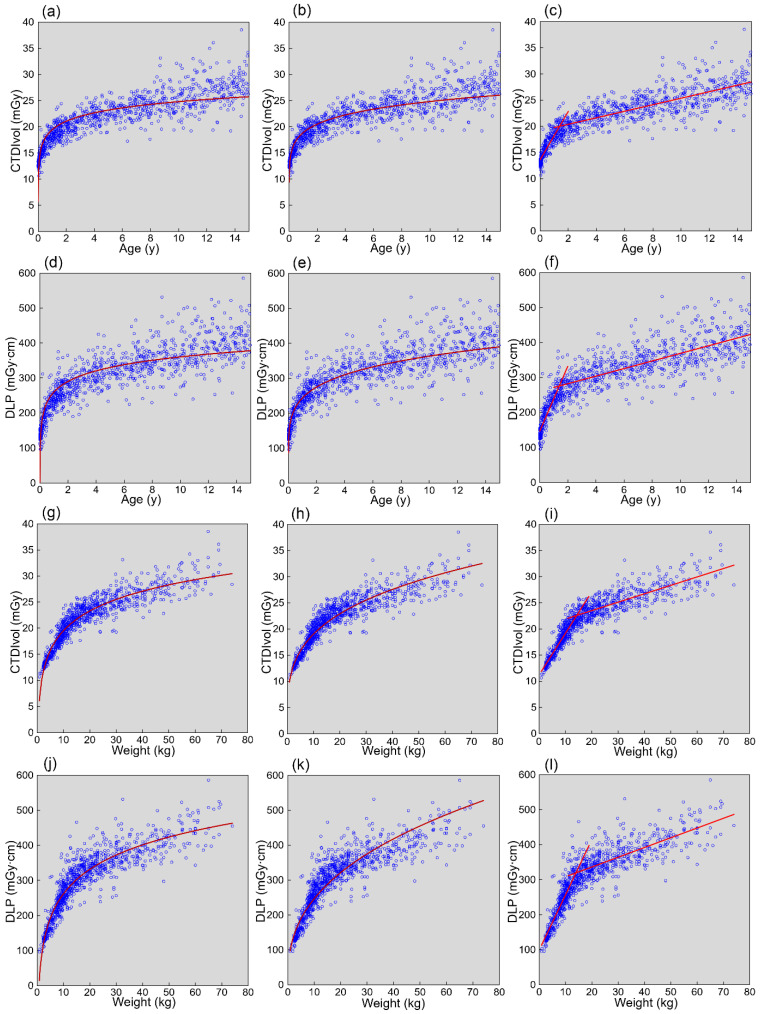
Relationships between CT dose indices and age or weight. Plots of CTDIvol versus age (**a**–**c**), DLP versus age (**d**–**f**), CTDIvol versus weight (**g**–**i**), and DLP versus weight (**j**–**l**) are presented. The red lines represent the fitting functions. Logarithmic (**a**,**d**,**g**,**j**), power (**b**,**e**,**h**,**k**), and bilinear functions (**c**,**f**,**i**,**l**) were used for fitting.

Similar to the mean effective diameter and mean water equivalent diameter, the CT dose indices, CTDIvol and DLP, increased with increasing age and weight, characterized by an initially rapid and subsequently slow increase (Figure 3). Logarithmic, power, and bilinear functions were well fitted to the plots of the CT dose indices against age and weight. The equations to estimate standard CT dose indices based on age and weight are presented in Table 1 and were used to estimate standard CT dose indices at representative ages (Table 2) and weights (Table 3). The age-based estimates just after birth (0.01 years) were apparently different depending on the type of fitting function.

**Figure 4:**
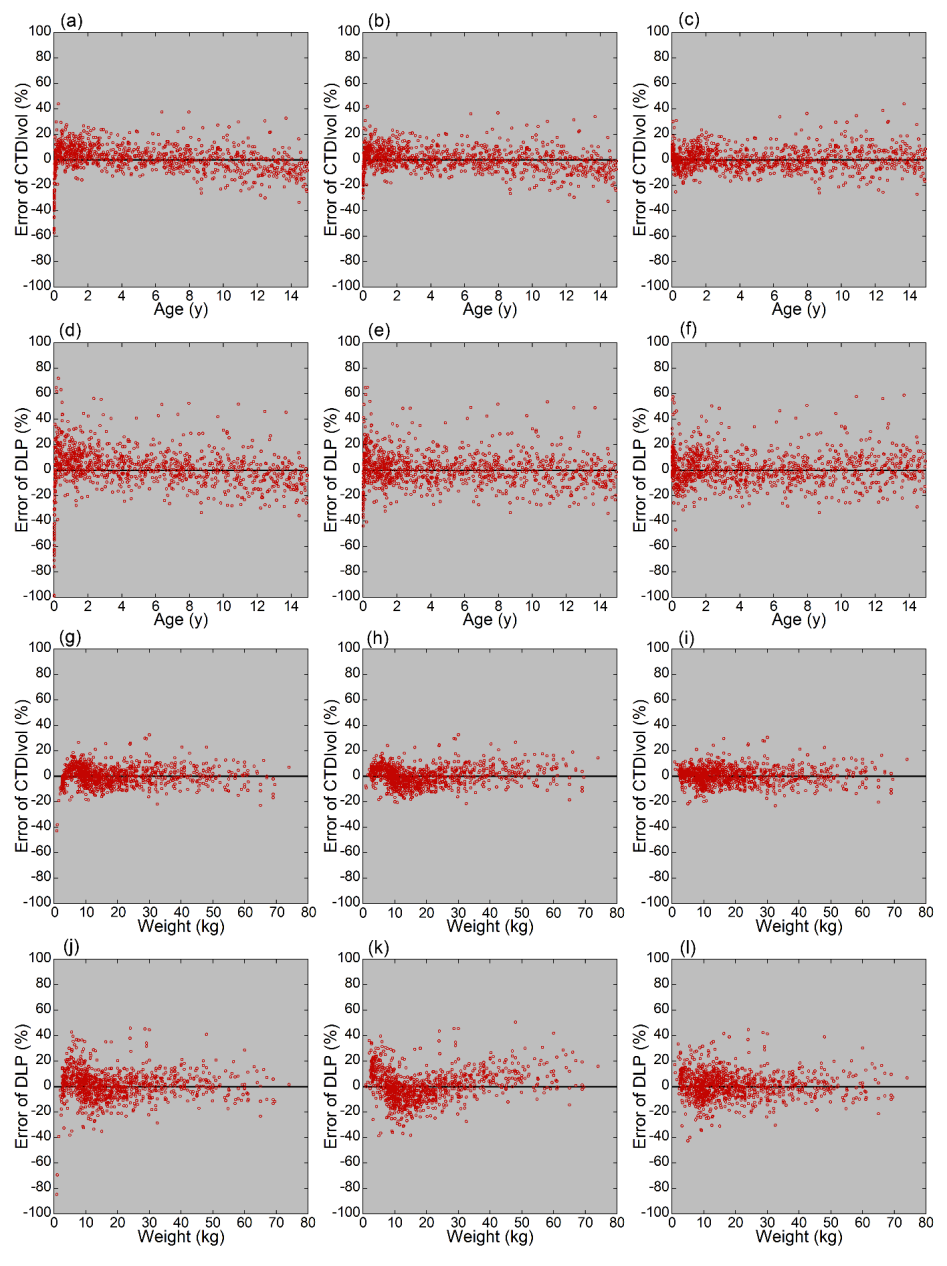
Relationships between errors and age or weight. Plots of error of CTDIvol versus age (**a**–**c**), error of DLP versus age (**d**–**f**), error of CTDIvol versus weight (**g**–**i**), and error of DLP versus weight (**j**–**l**) are presented. Logarithmic (**a**,**d**,**g**,**j**), power (**b**,**e**,**h**,**k**), and bilinear functions (**c**,**f**,**i**,**l**) were used for fitting.

When CTDIvol was estimated using the logarithmic function and age, the error was far below zero just after birth (Figure 4a), implying underestimation of CTDIvol. As a result, the SD of the error at 0–<0.5 years was large (18.7%, Table 4). With increasing age, the error deviated to the positive side approximately until 5 years, indicating a tendency toward overestimation of CTDIvol (Figure 4a, Table 4). The mean error became negative at 10–<15 years, indicating underestimation. When CTDIvol was estimated using the power function and age, the relationship between error and age resembled that observed using the logarithmic function (Figure 4b, Table 4), with relatively large variation at 0–<0.5 years, positive error at 0.5–<5 years, and negative error at 10–<15 years. However, the mean errors were closer to zero, regardless of age group, indicating a better fitting of the power function. When CTDIvol was estimated using the bilinear function and age, errors were distributed around zero regardless of age, and fitting was better than that of the other two functions (Figure 4c, Table 4). In age-based estimation, the errors were more variable for DLP (Figure 4d–f) than CTDIvol, as supported by the larger SD of the error for each age group (Table 4), implying a less precise estimation.

When CTDIvol was estimated using weight, the plots of errors were distributed in a narrower range around the horizontal axis (Figure 4g–i) than for the estimation using age, indicating a better estimation of standard CTDIvol. The SDs of the error were smaller for weight- (Table 5) than for age-based (Table 4) estimates. Although differences between the types of fitting functions were less evident for weight-based than for age-based estimation, differences in mean error among weight groups were the smallest for the bilinear function. Similar to the age-based estimation, the weight-based estimation caused larger variations in the errors of DLP (Figure 4j–l) than those of CTDIvol, as supported by the larger SD of the error for each weight group (Table 5).

In sex-based comparisons, the mean error of CTDIvol estimated using the bilinear function was negative in male patients and positive in female patients, indicating underestimation and overestimation of CTDIvol, respectively (Table 6). The sex-dependent difference was statistically significant for both age- and weight-based estimations (*p* < 0.0001). The error of DLP estimated using the bilinear function was also significantly larger in female than male patients, for both age-based and weight-based estimations (*p* < 0.0001).

## 4. Discussion

The growth of the head is rapid early after birth [18] and precedes that of the body. In the present study, the effective diameter and water equivalent diameter, indices of the section size used for CT, increased with increasing age, as reported previously in a small number of patients [19]. The increase was rapid approximately up to 1.5 years of age and slowed thereafter. Similarly, increases in these head size indices with increasing weight were less evident in larger than smaller patients, reflecting the precedence of head growth over body growth. In radiation dose management for pediatric brain CT, the biphasic alteration in head size and attenuation strength should be considered.

As children grow, more radiation exposure is required to obtain CT images of appropriate quality. In CT, the patient is exposed to X-ray photons from multiple directions, and photons passing through the patient are detected and used to reconstruct tomographic images. When the image section is larger, stronger attenuation decreases the proportion of X-ray photons reaching the detector, increasing image noise. Radiation exposure should, therefore, be increased to preserve image quality. Radiation output from an X-ray generator is proportional to tube current and is manipulated mainly by altering tube current. For optimization of radiation exposure according to patient size, a reference table may be prepared for the operator to determine the appropriate tube current depending on the age group. In automatic exposure control, the strength of attenuation is assessed mainly from the scout images, and the tube current is automatically modulated [20,21,22,23]. As the strength of attenuation differs among patients and among slice locations within a patient, automatic exposure control changes tube current according to the attenuation strength, even within an imaging series. This technology contributes to the optimization of radiation exposure for each patient and for each location.

In this study, 3D mA modulation was used for automatic exposure control. The relationships of CTDIvol and DLP with age and weight were investigated regarding age and weight as continuous variables. Similar to the effective diameter and water equivalent diameter, CTDIvol and DLP increased biphasically with age and weight. The similarity of the alteration patterns between head size indices and CT dose indices indicates appropriate modulation of tube current, corresponding to the strength of attenuation, using automatic exposure control. Automatic exposure control is not necessarily used in pediatric brain CT [24,25], but its use is recommended to optimize tube current according to head size. It should be noted that the relationships of CT dose indices with age and weight may differ depending on the imaging protocol and automatic exposure control software. In 3D mA modulation, the noise index is the main parameter defined by the user and determines the image noise level. In our clinical practice, the noise index is higher in the pediatric protocol (4) than in the adult protocol (3.3) to reduce radiation exposure in children; the resulting increase in image noise is considered acceptable. Although the noise index in this study was fixed irrespective of age, different noise indices may be selected between adult and pediatric patients, as well as among pediatric patients in different age groups, which may affect dose–age and dose–weight relationships. Tube current modulation also differs depending on the automatic exposure control software used [26,27,28,29]. Moreover, lowering tube potential has been shown to allow dose reduction, while preserving image quality, in pediatric brain CT [30]. Although a fixed tube potential was used in this study regardless of age, a lower tube potential may be used in smaller than larger children [25,31], possibly affecting dose–age and dose–weight relationships.

In the analysis of CT dose indices versus age, logarithmic, power, and bilinear functions were fitted to the plots, and equations were determined to estimate standard CT dose indices according to age. To assess the validity of the estimation using those equations, errors in the estimation were evaluated in relation to age. When CT dose indices were estimated using age and the logarithmic function, age-dependent discrepancies between estimated and actual values were noted, with underestimation just after birth, overestimation at 0.5–<5 years, and underestimation at 10–<15 years. These tendencies may be recognized by visual assessment of the dose–age plots with the fitting curve, as presented in Figure 3. Successful estimation is indicated by a fitting curve that passes through the center of the plots in the vertical direction regardless of age. Systematic age-dependent errors are undesirable in estimating standard CT dose indices according to age. Age-dependent errors were decreased using the power function and further decreased using the bilinear function. Therefore, a bilinear function or power function was suggested to be suitable for estimating CT dose indices from age.

Errors in the estimation of CT dose indices were mildly less variable using weight than age. Although weight-based grouping is recommended to define the DRL of body CT, the age-based grouping has been recommended for brain CT [12,13]. The results of this study suggest that, while age is acceptable, weight is preferable for radiation dose management in pediatric brain CT. As for the type of fitting function, while all three functions appeared to be acceptable, the best results were obtained with the bilinear function. Considering the results of the dose–age and dose–weight relationships together, fitting with a bilinear function appears recommendable for pediatric brain CT.

The variations in error were larger for DLP than for CTDIvol. DLP is an integral of CTDIvol over the axial scan range; it is calculated as a product of CTDIvol and scan length if CTDIvol is constant over the scan range. In a larger patient, both CTDIvol and scan length would increase, resulting in a larger increase in DLP than CTDIvol. The variability in DLP is derived from that in CTDIvol and, additionally, that in scan length, and inevitably exceeds that in CTDIvol.

In this study, data from male and female patients were pooled together, and the equations used to estimate standard CT dose indices were determined based on the entire dataset. When either age or weight was used, CT dose indices were underestimated in male patients and overestimated in female patients. This means that actual CT dose indices were larger in male than in female patients at a given age or weight. When sufficient data are available, separate equations for male and female patients would mildly improve estimation accuracy.

DRLs are established based on the standard CT dose indices reported by many imaging facilities in a country or region. To determine standard CT dose indices in each facility, data are usually collected for each age or weight group, and median values are defined as standard values [12,13]. However, there are problems with this process. The number of pediatric CT examinations is limited in many facilities; therefore, it is difficult to collect a sufficient volume of data for each age or weight group to determine the standard value with acceptable statistical validity [12,13,24]. Additionally, head size varies widely within a young group such as <1 year and 1–<5 years, and thus the distribution of age or weight within a group may affect the median value of the CT dose index. If the 1–5-year-old group includes many patients closer to 1 year of age, the median value may be low, whereas if the same age group includes many patients closer to 5 years of age, the median value may be higher. Narrowing the range of a group mitigates this effect but makes it more difficult to collect sufficient data for each group. Moreover, the age and weight ranges used for grouping are not fully standardized, which disturbs the comparison of different DRLs [32]. Curve fitting using age or weight as a continuous variable enables the determination of standard CT dose indices at any age or weight and allows estimation of standard values even in the age or weight range where CT is rarely performed in the facility [14,15].

The results of this study suggest that weight is preferable to age for determining standard dose indices at each facility. However, weight records are not always available [24,25,32], and the use of age is acceptable in case of lacking weight records. Although separate analyses of the data of male and female patients would improve the accuracy of determination of standard CT dose indices, DRLs are not defined for each sex, and analysis of data pooled from both sexes is also required. Although this study indicated that a bilinear function is preferable for curve fitting, an optimal fitting function may differ according to the imaging protocol. The fitting of bilinear, logarithmic, and power functions, followed by the selection of the optimal function based on visual evaluation of the fitting, may be recommended for each facility. Poor fit may suggest that the strength of radiation exposure is not well matched with head size. Visual evaluation of the plots and fittings may trigger an investigation for improvement of the imaging protocol.

Using equations determined from the data of each imaging facility, standard CT dose indices at given ages and weights can be calculated as presented in Table 2 and Table 3. For routine quality assessment, deviation of the dose index of every examination from the standard dose estimated based on the patient’s weight or age may be calculated, followed by the extraction of examinations with a large deviation and analysis of the cause of the deviation. When establishing DRLs, each facility will determine equations for estimation and calculate standard dose indices at each age or weight, as designated by the authority responsible for establishing the DRLs. The authority collects standard dose indices from many facilities and determines the DRL value as the 75th percentile of the dose distribution. Differences in the type of fitting function among facilities do not matter because the values calculated at given ages or weights, but not the equations themselves, are compared.

For radiation dose management of brain CT in children, CT dose indices, CTDIvol, and DLP are usually evaluated for each age group. In this study, using a large volume of pediatric brain CT data, logarithmic, power, and bilinear functions were well fitted to the plots of the CT dose indices against age and weight, allowing estimation of standard dose indices at any age or weight. Error analysis suggested that weight was mildly better than age and that the best results were obtained with a bilinear function. These findings are expected to contribute to the improvement of radiation dose management in pediatric brain CT. However, there are limitations to this study. First, data obtained in a single facility were analyzed. The relationship between CT dose indices and age or weight may depend on the patient population, CT scanner, and imaging protocols, and therefore, validation in other facilities should be important. Next, a much smaller volume of data will be used in actual radiation dose management, and the uncertainty of curve fitting in such a situation is a subject of future research. As for bilinear fitting, we subjectively set the cutoffs as 1.5 years and 15 kg. Optimal cutoffs and the influence of cutoff selection on the estimation of standard dose indices remain to be investigated. Additionally, although the balance between radiation dose and image quality should be considered for optimization in medical imaging, we did not assess image quality. Evaluation of the appropriateness of our imaging protocol is beyond the scope of this study.

## 5. Conclusions

Curve fitting of the relationship between CT dose indices and age or weight as continuous variables facilitates the determination of standard dose indices in pediatric brain CT at each facility. Weight provides better estimates than age, and a bilinear function is preferable for curve fitting. The method proposed here for determining standard CT dose indices is expected to aid the establishment and application of DRLs for pediatric brain CT.

## Figures and Tables

**Table 1:** Equations used to estimate CT dose indices.

Variable	Estimate	Logarithmic	Power	Bilinear	
				Young/Small	Old/Large
Age	CTDIvol	y = 2.328 ln(x) + 19.46	y = 18.888x^0.1188^	y = 4.620x + 13.62	y = 0.625x + 19.13
Age	DLP	y = 43.70 ln(x) + 259.9	y = 243.46x^0.1738^	y = 92.83x + 146.6	y = 10.85x + 260.5
Weight	CTDIvol	y = 5.467 ln(x) + 6.92	y = 10.261x^0.2680^	y = 0.7941x + 11.17	y = 0.1609x + 20.28
Weight	DLP	y = 100.63 ln(x) + 29.9	y = 103.25x^0.3791^	y = 15.791x + 99.0	y = 2.787x + 280.2

CTDIvol, DLP, age, and weight are expressed in units of mGy, mGy∙cm, y, and kg, respectively.

**Table 2:** CT dose indices estimated from age.

Age (y)	CTDIvol (mGy)			DLP (mGy∙cm)		
	Logarithmic	Power	Bilinear	Logarithmic	Power	Bilinear
0.01	8.7	10.9	13.7	59	109	147
0.5	17.8	17.4	15.9	230	216	193
1	19.5	18.9	18.2	260	243	239
5	23.2	22.9	22.3	330	322	315
10	24.8	24.8	25.4	360	363	369
15	25.8	26.1	28.5	378	390	423

**Table 3:** CT dose indices estimated from weight.

Weight (kg)	CTDIvol (mGy)			DLP (mGy∙cm)		
	Logarithmic	Power	Bilinear	Logarithmic	Power	Bilinear
3	12.9	13.8	13.6	140	157	146
5	15.7	15.8	15.1	192	190	178
10	19.5	19.0	19.1	262	247	257
20	23.3	22.9	23.5	331	321	336
40	27.1	27.6	26.7	401	418	392
60	29.3	30.7	29.9	442	488	447

**Table 4:** CT dose indices estimated from age.

Age (y)	*n*	Error of CTDIvol (%)			Error of DLP (%)		
		Logarithmic	Power	Bilinear	Logarithmic	Power	Bilinear
0–<0.5	152	−3.1 ± 18.7	0.7 ± 11.9	1.1 ± 7.8	−3.3 ± 34.0	4.2 ± 19.6	3.7 ± 15.9
0.5–<1	59	7.3 ± 8.1	4.3 ± 7.9	−2.0 ± 7.5	9.2 ± 11.7	2.3 ± 11.0	−4.0 ± 10.7
1–<2	128	5.6 ± 8.0	2.6 ± 7.8	2.6 ± 7.9	6.9 ± 11.9	0.5 ± 11.2	4.4 ± 11.7
2–<5	185	4.9 ± 8.1	2.7 ± 7.8	0.3 ± 7.9	6.2 ± 13.2	2.0 ± 12.5	1.4 ± 12.8
5–<10	239	1.1 ± 8.7	0.4 ± 8.7	−0.2± 8.7	1.7 ± 12.2	0.9 ± 12.1	0.0 ± 12.1
10–<15	217	−4.4 ± 10.5	−3.8 ± 10.5	1.7 ± 10.8	−4.2 ± 13.2	−2.4 ± 13.3	2.4 ± 13.8
Total	980	0.9 ± 11.8	0.5 ± 9.7	0.8 ± 8.8	1.6 ± 18.3	0.9 ± 13.8	1.7 ± 13.3

**Table 5:** CT dose indices estimated from weight.

Weight (kg)	*n*	Error of CTDIvol (%)			Error of DLP (%)		
		Logarithmic	Power	Bilinear	Logarithmic	Power	Bilinear
0–<5	100	−2.3 ± 8.4	3.5 ± 5.3	1.8 ± 5.1	1.1 ± 16.5	12.0 ± 11.5	5.3 ± 11.0
5–<10	199	3.5 ± 6.6	1.7 ± 6.7	−0.4 ± 6.1	5.4 ± 12.8	0.5 ± 12.5	−0.3 ± 11.7
10–<15	191	−0.7 ± 6.5	−3.2 ± 6.4	0.5 ± 6.8	−1.8 ± 10.5	−7.0 ± 9.9	1.3 ± 11.0
15–<20	118	−1.9 ± 6.7	−3.9 ± 6.6	0.8 ± 6.9	−2.0 ± 9.8	−6.0 ± 9.5	1.9 ± 10.3
20–<40	255	1.0 ± 7.5	0.6 ± 7.6	0.1 ± 7.4	1.6 ± 10.6	1.4 ± 11.0	0.3 ± 10.4
40–<80	117	0.7 ± 7.8	4.2 ± 7.8	1.1 ± 7.6	2.0 ± 10.0	9.8 ± 10.5	1.5 ± 9.8
Total	980	0.5 ± 7.4	0.3 ± 7.4	0.5 ± 6.8	1.3 ± 11.9	0.8 ± 12.6	1.2 ± 10.8

**Table 6:** Errors for each sex.

Variable	Error of CTDIvol (%)		Error of DLP (%)	
	Male	Female	Male	Female
Age	−1.2 ± 8.2	3.2 ± 9.0	−1.2 ± 11.9	5.3 ± 14.0
Weight	−0.9 ± 6.3	2.2 ± 7.0	−0.9 ± 9.7	3.8 ± 11.6

## Data Availability

The data are available upon reasonable request from the corresponding author.
